# Transcriptional Regulation of *opaR*, *qrr2–4* and *aphA* by the Master Quorum-Sensing Regulator OpaR in *Vibrio parahaemolyticus*

**DOI:** 10.1371/journal.pone.0034622

**Published:** 2012-04-10

**Authors:** Yiquan Zhang, Yefeng Qiu, Yafang Tan, Zhaobiao Guo, Ruifu Yang, Dongsheng Zhou

**Affiliations:** 1 State Key Laboratory of Pathogen and Biosecurity, Beijing Institute of Microbiology and Epidemiology, Beijing, China; 2 Laboratory Animal Center, Academy of Military Medical Sciences, Beijing, China; National Institutes of Health, United States of America

## Abstract

**Background:**

*Vibrio parahaemolyticus* is a leading cause of infectious diarrhea and enterogastritis via the fecal-oral route. *V. harveyi* is a pathogen of fishes and invertebrates, and has been used as a model for quorum sensing (QS) studies. LuxR is the master QS regulator (MQSR) of *V. harveyi*, and LuxR-dependent expression of its own gene, *qrr2–4* and *aphA* have been established in *V. harveyi*. Molecular regulation of target genes by the *V. parahaemolyticus* MQSR OpaR is still poorly understood.

**Methodology/Principal Findings:**

The bioinformatics analysis indicated that V. *parahaemolyticus* OpaR, *V. harveyi* LuxR, *V. vulnificu* SmcR, and *V. alginolyticus* ValR were extremely conserved, and that these four MQSRs appeared to recognize the same conserved *cis*-acting signals, which was represented by the consensus constructs manifesting as a position frequency matrix and as a 20 bp box, within their target promoters. The MQSR box-like sequences were found within the upstream DNA regions of *opaR*, *qrr2–4* and *aphA* in *V. parahaemolyticus*, and the direct transcriptional regulation of these target genes by OpaR were further confirmed by multiple biochemical experiments including primer extension assay, gel mobility shift assay, and DNase I footprinting analysis. Translation and transcription starts, core promoter elements for sigma factor recognition, Shine-Dalgarno sequences for ribosome recognition, and OpaR-binding sites were determined for the five target genes of OpaR, which gave a structural map of the OpaR-dependent promoters. Further computational promoter analysis indicated the above regulatory circuits were shared by several other closely related Vibrios but with slight exceptions.

**Conclusions/Significance:**

This study gave a comprehensive computational and characterization of the direct transcriptional regulation of five target genes, *opaR*, *qrr2–4* and *ahpA*, by OpaR in *V. parahaemolyticus*. These characterized regulatory circuits were conserved in *V. harveyi* and *V. parahaemolyticus*.

## Introduction

Bacterial quorum sensing (QS) is a process of population density-dependent cell-to-cell communication through synthesizing, releasing, and detecting signal molecules called autoinducers. The QS systems are widely distributed in *Vibrio* species that are natural inhabitants in seawater, boundary regions between sea and river, and aquatic products. *V. harveyi* (a pathogen of fishes and invertebrates) and *V. cholerae* (the causative agent of the disease cholera) have been used as models for QS studies (summarized or characterized in [1], [2], [3], [4], [5], [6], [7], [8], [9], [10], [11], [12], [13]).

**Figure 1:**
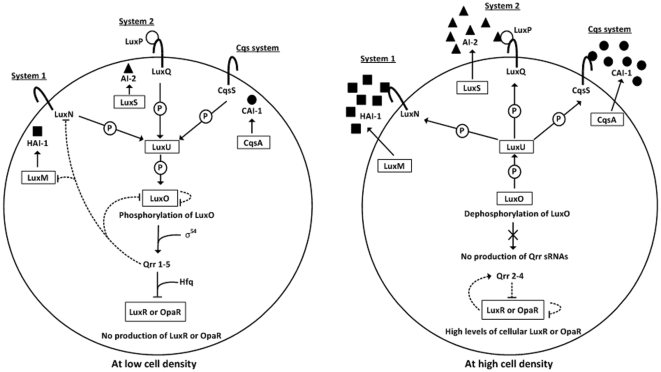
Quorum sensing systems in *V. harveyi*/*V. parahaemolyticus*. The mode for signal transduction during QS in *V. harveyi* has been described in the text. The feedback regulatory loops are shown with dotted lines. Since all the components of *V. harveyi* quorum sensing appears to be intact in the *V. parahaemolyticus* genome [16], the QS signal transduction cascades should be conserved in *V. harveyi* and *V. parahaemolyticus*.

*V. harveyi* uses three distinct autoinducers, harveyi autoinducer 1 (HAI-1), autoinducer 2 (AI-2), and cholerae autoinducer 1 (CAI-1), which are synthesized by the autoinducer synthases LuxM, LuxS, and CqsA, respectively (Fig. 1). They bind to the membrane-anchoring receptor proteins, LuxN, LuxP/LuxQ, and CqsS, respectively, at the cell surface. *V. cholerae* uses two known autoinducers CAI-1 and AI-2, rather than HAI-1, since the orthologs of LuxN and LuxM are essentially absent from this bacterium. The association of autoinducers and their receptor proteins triggers a common phosphorylation/dephosphorylation signal transduction cascade involving LuxU and LuxO.

At low cell density (LCD) (Fig. 1), the autoinducers are absent or at low concentrations, and the receptors autophosphorylate and then transfer phosphate to the phosphorelay protein LuxU that in turn shuttles phosphate to the transcriptional factor LuxO. The phosphorylated LuxO (LuxO-P) in combination with the sigma factor σ^54^ activates the transcription of the genes encoding regulatory small RNAs (sRNAs), Qrr1–4. The Qrr sRNAs accompanying with the RNA-binding protein Hfq in turns inhibit the translation of the mRNA of the master QS regulator (MQSR), e.g., LuxR in *V. harveyi*, and HapR in *V. cholerae*, which leads to the ceased production of MQSR. The over-production of Qrr sRNAs and LuxO-P triggers three feedback regulatory loops: i) LuxO-P represses the transcription of its own gene, ii) Qrr sRNAs inhibits the translation of *luxO*, and iii) Qrr sRNAs repress the translation of *luxMN* encoding LuxM and its cognate receptor LuxM; these feedbacks will contribute to control the Qrr levels within physiological states, since the *qrr* expression requires the signal transduction among LuxO, LuxM, and LuxN.

At high cell densities (HCD) (Fig. 1), the accumulated autoinducers bind to their receptors, and convert the receptors to phosphatases, thereby reversing the phosphate flow and triggering the dephosphorylation of LuxU and LuxO. Dephosphorylated LuxO cannot activate the *qrr* transcription. Existing Qrr sRNAs are rapidly turned over, as Hfq-dependent sRNAs are degraded stoichiometrically with their target mRNAs. In the absence of the Qrr sRNAs, the MQSR mRNA is translated, and MQSR is produced abundantly to act as either a transcriptional activator or a repressor for its target genes. Overproduced MQSR will feed back to repress its own transcription.

MQSR is able to activate the transcription of *qrr* genes, and this MQSR-*qrr* feedback loop (Fig. 1) in turns leads to the rapid down-regulation of MQSR gene. The efficient stimulation of *qrr* genes requires the simultaneous presence of LuxO-P (abundant only at LCD) and MQSR (abundant only at HCD). LuxO-P and MQSR are thought to be simultaneously present immediately following the switch from HCD to LCD. Therefore, the above negative feedback loop dramatically accelerates the transition HCD to LCD, but it has no effect on the QS behaviors at steady-state LCD or HCD.

The above five negative feedback loops control the integration of multiple signals, and maintain the signal transmission fidelity of QS through affecting the input-output dynamic range of signal transmission and modulating the noise in the output [13].

*V. parahaemolyticus* is a leading cause of infectious diarrhea and enterogastritis via the fecal-oral route [14]. Human infections occur mainly due to the ingestion of this pathogen in raw or undercooked seafood. *V. harveyi*, *V. parahaemolyticus*, and six additional closely related species (*V. alginolyticus*, *V. campbellii*, *V. rotiferianus*, *V. natrigens*, *V. mytili*, and *V. azureus*) constitute the Harveyi clade that is a subset of the *Vibrio* core group [15]. All the components of the *V. harveyi* QS system can be annotated to be intact in the genome *V. parahaemolyticus* [16]. Thus, the signal transduction cascades of QS should be conserved in *V. harveyi* and *V. parahaemolyticus* (Fig. 1).

The *V. parahaemolyticus* OpaR [17] is the ortholog of the *V. harveyi* LuxR. The *opaR* gene (VP2516) [16] is composed of an open reading frame (ORF) containing 615 nucleotides with a G+C content of 44.55%, and it encodes a deduced protein of 204 amino acids (a.a.) with a calculated molecular mass of 23634.01 Da and with an isolectric point of 5.96. Regulation of target genes by OpaR in *V. parahaemolyticus* is still poorly understood. In the present work, the consensus constructs were built to represent the conserved *cis*-acting signals recognized by the four extremely conserved MQSR proteins, *V. parahaemolyticus* OpaR, *V. harveyi* LuxR, *V. vulnificus* SmcR, and *V. alginolyticus* ValR, which was followed by a comprehensive molecular characterization of the transcriptional regulation of five target genes, *opaR*, *qrr2–4* and *ahpA*, by OpaR in *V. parahaemolyticus*.

## Materials and Methods

### Bacterial strains

The wild-type *V. parahaemolyticus* strain RIMD 2210633 (WT) is a pandemic O3:K6 strain isolated from a patient with traveler's diarrhea in Japan in 1996 [16]. The null *opaR* mutant derived from WT and the corresponding complemented mutant are described below. For the common bacterial growth and maintenance, bacteria were cultured in Luria-Bertani (LB) broth or LB agar with addition of 2% NaCl at 37°C, and chloramphenicol was added at 5 µg/ml when needed. For the longtime storage, bacteria were stored in Difco™ Marine (MR) broth 2216 (BD Bioscience) with addition of 30% glycerol at −85°C.

### Construction of the *opaR* null mutant

The entire coding region of *opaR* was deleted from RIMD 2210633 to generate the *opaR* null mutant strain *ΔopaR*, using the suicide plasmid pDS132 [18] by introducing homologous recombination as previously described [19], [20]. Briefly, the 414 and 457 bp DNA regions upstream and downstream of *opaR*, respectively, were amplified by PCR, purified, and used as the templates to create a 871 bp deletion construct, through PCR, which was subsequently inserted between the *Pst*I and *Sph*I sites of pDS132. This generated a recombinant vector that contained the deletion construct and the *sacB* gene conferring sensitivity to sucrose. All the primers used in the present work were listed in Table 1. Upon verification by DNA sequencing, the recombinant vector was introduced into *Escherichia coli* S17-1(pir), and then transferred into RIMD 2210633 by conjugation. The mutant strain was selected using resistance to 10% sucrose and sensitivity to chloramphenicol, and further verified by PCR.

**Table 1:** Oligonucleotide primers used in this study.

Target	Primers (forward/reverse, 5′-3′)
**Construction of mutant**	
*opaR*	GTGACTGCAGACTGCCTTGGTAACGCTCTG/GTTCGTGTTCAAATCTGAGCTATCCATTTTCCTTGCCATTTG
	CAAATGGCAAGGAAAATGGATAGCTCAGATTTGAACACGAAC/GTGAGCATGCATGGGCTGCATCAGGTCG
	GTGACTGCAGACTGCCTTGGTAACGCTCTG/GTGAGCATGCATGGGCTGCATCAGGTCG
**Complementation of mutant**	
*opaR*	GCGGGATCCTCCATCGTGTTGCCGTAGC/GCGAAGCTTGCGAAAGCAGAAGGCATCAAG
**Protein expression**	
*opaR*	AGCGGGATCCATGGACTCAATTGCAAAGAG/AGCGAAGCTTTTAGTGTTCGCGATTGTAG
**EMSA**	
*opaR*	TGTGGGTTGAGGTAGGTCG/GCCTAGTTCTAGGTCTCTTTGC
*qrr2*	AGTGGTTGCTTATGAATC/GGTCGAGAAGTATTATGC
*qrr3*	GGATAAGTTCAAATTGGATC/GTGGTTTCTGTGACATAC
*qrr4*	AACCGTGAAATCCATTTAC/CGACGCATTATTAACCAG
*aphA*	AACTTCCAACCACATAATTGCG/GGCTGGAGCAGGTATGATTG
**DNase I footprinting**	
*opaR*	AGTGGGTTGAAAGTCACATCC/GCCTAGTTCTAGGTCTCTTTGC
*qrr2*	AGTGGTTGCTTATGAATC/GGTCGAGAAGTATTATGC
*qrr3*	GGATAAGTTCAAATTGGATC/GTGGTTTCTGTGACATAC
*qrr4*	AACCGTGAAATCCATTTAC/CGACGCATTATTAACCAG
*aphA*	AACTTCCAACCACATAATTGCG/GGCTGGAGCAGGTATGATTG
**Primer extension**	
*opaR*	/ATCCATTTTCCTTGCCATTTG
*qrr2*	/TTATTGTGAACAATCTATAT
*qrr3*	/AATCAAGTTCACTAACAAC
*qrr4*	/ATATACTTGTGAACAATGTG
*aphA*	/GCTCTTACTGGCGCTTGAG

### Complementation of *ΔopaR*

For complementation of the *ΔopaR* mutant, a PCR-generated DNA fragment containing the *opaR* coding region together with its promoter region (539 bp DNA region upstream of the coding sequence) and transcriptional terminator region (327 bp DNA region downstream) were cloned between the *Sal*I and *Sph*I sites of the vector pBRMob (kindly proved by Prof. Hin-chung Wong from Taiwan Soochow University) which is the ligation product of a 3219 bp fragment (containing the RP4 mob DNA region for plasmid mobilization) from pDS132 digested with *Hind*III, and the *Hind*III-digested plasmid pBR328 (harboring a chloramphenicol resistance gene) [21]. The recombinant plasmid, verified by DNA sequencing, was subsequently introduced into *ΔopaR*, yielding the complemented mutant strain *C-opaR*.

### Bacterial growth and RNA isolation

The glyceric stock of bacterial cells was inoculated into 5 ml of the MR broth for growing at 30°C with shaking at 200 rpm for 24 h. The cell culture was 40-fold diluted with the PBS buffer (pH 7.2), and then 150 µl of the diluted cells were spread onto a HI plate [2.5% Bacto heart infusion (BD Bioscience), and 1.5% bacteriological grade agar] with a diameter of 5 cm. After 8 h of growth at 30°C, cells were harvested from the plate by adding the mixture of 1.5 ml of RNAprotect (Qiagen) and 0.5 ml of PBS. Bacterial cultivations were done at least in triplicate (at least three biological replicates) for each strain.

Total bacterial RNAs were extracted using the TRIzol Reagent (Invitrogen) [22], [23], [24]. RNA quality was monitored by agarose gel electrophoresis, and RNA quantity was determined by spectrophotometry.

### Primer extension assay

For the primer extension assay [22], [23], [24], an oligonucleotide primer complementary to a portion of the RNA transcript of each indicated gene was employed to synthesize cDNAs from the RNA templates. About 10 µg of the total RNA from each strain was annealed with 1 pmol of [γ-^32^P] end-labeled reverse primer using a Primer Extension System (Promega) according to the manufacturer's instructions. The same labeled primer was also used for sequencing with the fmol® DNA Cycle Sequencing System (Promega). The primer extension products and sequencing materials were concentrated and analyzed in a 6% polyacrylamide/8 M urea gel. The result was detected by autoradiography (Kodak film).

### Preparation of purified OpaR protein

Preparation of the purified OpaR protein was performed as previously described [22], [23], [24]. The entire coding region of the *opaR* gene of strain RIMD 2210633 was directionally cloned between the *BamH*I and *Hind*III sites of plasmid pET28a (Novagen). The recombinant plasmid encoding the 6× His-tagged OpaR protein (His-OpaR) was transformed into *Escherichia coli* BL21λDE3 cells. Expression of His-OpaR was induced by the addition of 1 mM IPTG (isopropyl-b-D-thiogalactoside). The overproduced protein was purified under native conditions using an Ni-NTA Agarose Column (Qiagen). The purified protein was concentrated with the Amicon Ultra-15 centrifugal filter device (Millipore), and the protein purity was verified by SDS-PAGE.

### Gel mobility shift assay (EMSA)

The 300 to 600 bp promoter-proximal region of each indicated gene was amplified by PCR. For EMSA [22], [23], [24], the 5′ ends of DNA were labeled using [γ-^32^P] ATP and T4 polynucleotide kinase. DNA binding was performed in a 10 µl reaction volume containing binding buffer [1 mM MgCl_2_, 0.5 mM EDTA, 0.5 mM DTT, 50 mM NaCl, 10 mM Tris-HCl (pH 7.5) and 0.05 mg/ml poly-(dI-dC)], labeled DNA (1000 to 2000 c.p.m/µl), and increasing amounts of the His-OpaR protein. Three controls were included in each EMSA experiment: 1) cold probe as specific DNA competitor (the same promoter-proximal DNA region unlabeled), 2) negative probe as nonspecific DNA competitor (the unlabeled coding region of the 16S rRNA gene), and 3) nonspecific protein competitor [rabbit anti-F1-protein polyclonal antibodies]. The F1 protein is the protective antigen from *Yersinia pestis* [25]. After incubation at room temperature for 30 min, the products were loaded onto a native 4% (w/v) polyacrylamide gel, and electrophoresed in 0.5× TBE buffer for about 50 min at 220 V. Radioactive species were detected by autoradiography after exposure to Kodak film at −70°C.

### DNase I footprinting

For DNase I footprinting [22], [23], [24], the 250 to 600 bp promoter-proximal DNA regions with a single ^32^P-labeled end were PCR amplified with either the sense or antisense primer being end-labeled. The PCR products were purified using the QiaQuick columns (Qiagen). Increasing amounts of His-OpaR were incubated with the purified, labeled DNA fragment (2 to 5 pmol) for 30 min at room temperature, in a final 10 µl reaction volume containing the binding buffer used in EMSA. Before DNA digestion, 10 µl of Ca^2+^/Mg^2+^ solution (5 mM CaCl_2_ and 10 mM MgCl_2_) was added, followed by incubation for 1 min at room temperature. The optimized RQ1 RNase-Free DNase I (Promega) was then added to the reaction mixture, and the mixture was incubated at room temperature for 40 to 90 s. The reaction was quenched by adding 9 µl of stop solution (200 mM NaCl, 30 mM EDTA, and 1% SDS), followed by incubation for 1 min at room temperature. The partially digested DNA samples were extracted with phenol/chloroform, precipitated with ethanol, and analyzed in 6% polyacrylamide/8 M urea gel. Protected regions were identified by comparison with the sequence ladders. For sequencing, we used the *fmol*® DNA Cycle Sequencing System (Promega). The templates for sequencing were the same as the DNA fragments of DNase I footprinting assays. Radioactive species were detected as previously described.

### Computational promoter analysis

The 300 bp promoter regions upstream of the start codon of each indicated gene were retrieved with the ‘*retrieve-seq*’ program [26]. Known or predicted binding sites of OpaR and its orthologs were collected and aligned to generate the position frequency matrix (PFM) by using the ‘*matrices-consensus*’ tool [26].The sequence logo representation of the above binding sites was generated by the WebLogo tool [27]. The ‘*matrices-paster*’ tool [26] was used to match the PFM within the promoter-proximal DNA regions.

## Results

### Phylogeny of OpaR and its orthologs

**Figure 2:**
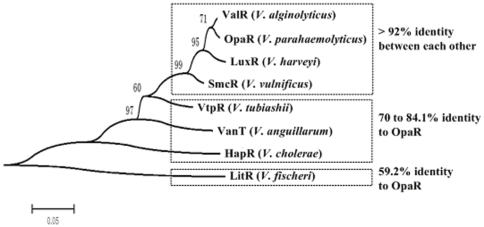
Phylogenetic tree of OpaR and its orthologs. Protein sequences were derived from *V. alginolyticus* ZJ-51 [60], *V. parahaemolyticus* RIMD 2210633 [16], *V. harveyi* ATCC BAA-1116 [61], *V. vulnificus* YJ016 [62], *V. tubiashii* RE22 [63], *V. anguillarum* 775 [64], *V. cholera* N16961 [65], and *V. fischeri* MJ11 [66]. The a.a. sequences were aligned by the CLUSTALW [67] web server at http://align.genome.jp/. The aligned sequences were then used to construct an unrooted neighbor-joining tree using the MEGA version 5.0 [68] with a bootstrap iteration number of 1000. Shown on the branch points of phylogenic tree were the bootstrap values (%).

The OpaR regulator shares high identity (≥70%) in a.a. sequences with the orthologous MQSRs in other six *Vibrio* species tested (*V. alginolyticus* ValR, *V. harveyi* LuxR, *V. vulnificus* SmcR, *V. tubiashii* VtpR, *V. anguillarum* VanT, and *V. cholerae* HapR). A phylogenetic tree (Fig. 2) was constructed from the aligned a.a. sequences of the above seven orthologous MQSR proteins, with an additional regulator LitR from *V. fischeri* [28] as the outgroup (LitR has about 60% identity to the above seven MQSRs; all these eight proteins are belonged to the TetR-family DNA-binding regulators), which revealed that LuxR, OpaR, SmcR, and ValR constituted the most closely related group (>92% identity between each other).

### The MQSR consensus

**Figure 3:**
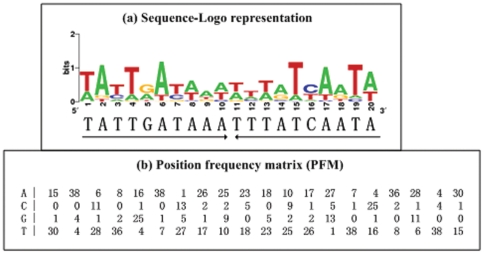
MQSR consensus constructs. (a) The sequence logo representation of the binding sites of OpaR and its orthologs (Table 2) was generated by the WebLogo tool [27]. The 20-bp consensus box TATTGATAAA-TTTATCAATA was annotated as an inverted repeat sequence. (b) A position frequency matrix describes the alignment of the binding sites, and denotes the frequency of each nucleotide at each position.

Since the four DNA-binding regulatory proteins LuxR, OpaR, SmcR, and ValR were extremely conserved, they should recognize the same conserved signals within their target promoters in *V. harveyi*, *V. parahaemolyticus*, *V. vulnificus*, and *V. alginolyticus*. Known or predicted binding sites of LuxR, OpaR, SmcR, and ValR (Table 2) were collected, and then aligned to generate the MQSR consensus that manifested as a PFM (in which each row and column represents a position and a nucleotide, respectively) and as a 20 bp invert-repeat sequence TATTGATAAA-TTTATCAATA termed as the MQSR box (Fig. 3).

**Table 2:** Known or predicted direct targets of OpaR or its orthologs.

Gene name	Gene ID	Consensus-like sequence	Position&	Score	Reference	Regulation
***V. harveyi*** ** ATCC BAA-1116**						
*luxR*	VIBHAR_03459	TAATGACATTACTGTCTATA	D-71…-52	9.99	[29]	$
		AACTATTAAAATAATCAATT	D-152…-133	10.93	[29]	$
*qrr2*	VIBHAR_04846	TGATGATTTATTTATCACTT	D-165…-146	7.62	[7]	#
*qrr3*	VIBHAR_05322	AGTTAATTAATTCATCATTA	D-151…-132	8.02	[7]	#
*qrr4*	VIBHAR_06697	TTCTGATAAATGTATTAGTA	D-167…-148	9.34	[7]	#
*aphA*	VIBHAR_00046	TATTGAGTATTTTATTAGTT	D-281…-262	12.04	[30]	$
*exsB*	VIBHAR_01694	TTTTAATAAAAAGATAAGTA	D-135…-116	6.45	[31]	$
*qrgB*	VIBHAR_00176	TATTGATTGTGAACTCAATA	D-98…-79	10.25	[3], [30]	$
*luxC*	VIBHAR_06244	TACAAATAACATTAATAATT	D-275…-256	8.49	[58]	#
		TATAAATAAATCAAACTATA	D-151…-132	8.88	[58]	#
*argA*	VIBHAR_03295	AATTGAATAAGAAGACAATA	D-64…-45	6.6	[59]	#
***V. parahaemolyticus*** ** RIMD 2210633**						
*opaR*	VP2516	TAATGACATTACTGTCTATA	D-149…-130	9.99	This study	$
		AATTATTAAAATAATCAATT	D-68…-49	11.29	This study	$
*qrr2*	VPA1623–1624 intergenic	GACTAACTCAATTGTTAATA	D-167…-148	7.8	This study	#
*qrr3*	VPA1240–1241 intergenic	TGTTTATTAATCAATCATTA	D-150…-131	8.5	This study	#
*qrr4*	VPA0199–0200 intergenic	TGCTGAGAAAGTGATTAGTA	D-167…-148	8.16	This study	#
*aphA*	VP2762	TATTGAGTATTATGTTAGTT	D-279…-260	10.68	This study	$
***V. vulnificus*** ** YJ016**						
*smcR*	VV2770	TATTGACATTACTGTTCATT	D-158…-139	8.56	Predicted	$
		AATTATTAAAACAATCAATA	D-78…-59	11.33	Predicted	$
*qrr3*		TATAAATAGATTTATTATTA	D-191…-172	10.79	Predicted	#
*aphA*	VV3005	TATTGAGCATTTTGTTAGTT	D-278…-259	10.50	Predicted	$
***V. alginolyticus*** ** ZJ-51**						
*valR*		TAATGACATTACTGTATATA	D-146…-127	7.60	Predicted	$
		AACTATTAAAATAATCAATT	D-65…-46	10.93	Predicted	$

& , ‘D’ indicates the direct sequence, and minus numbers denote nucleotide positions upstream of genes.

Negative ($) or positive (#) regulation by LuxR or its relevant ortholog.

LuxR-dependent expression of its own gene [29], *qrr2–4* [7], and *aphA* [30] have been established in *V. harveyi*. The presence of MQSR box-like sequences within the upstream DNA regions of the corresponding target genes in *V. parahaemolyticus*, as revealed by the computational promoter analysis (Table 2), indicated that the above regulatory cascades were conservatively controlled by the LuxR ortholog OpaR in *V. parahaemolyticus*, which were further validated by the following biochemical experiments.

### Mutation and complementation of *opaR*

Real-time RT-PCR experiments were performed to assess the relative mRNA levels of *opaR* in WT, *ΔopaR*, and *C-opaR*; the *opaR* transcript was lacking in *ΔopaR*, but was restored in *C-opaR* relative to WT (data not shown), indicating the successful mutation of *opaR* and the complementation of the *opaR* mRNA level.

As determined by several distinct methods (see below), the *ahpA* gene was negatively regulated by OpaR. To test whether the *opaR* mutation had the polar effect, the primer extension assays were conducted to detect the yield of the primer extension product of *ahpA* that represented the *ahpA* mRNA levels in WT, *ΔopaR*, and *C-opaR*. Herein, the *ahpA* mRNA level was significantly enhanced in *ΔopaR* relative to WT, while no obvious change in the *ahpA* transcription was observed between WT and *C-opaR* (Fig. S1). This analysis confirmed that the detecting enhanced transcription of *ahpA* in *ΔopaR* was due to the *opaR* mutation rather than a polar effect.

### Growth of WT and *ΔopaR*

**Figure 4:**
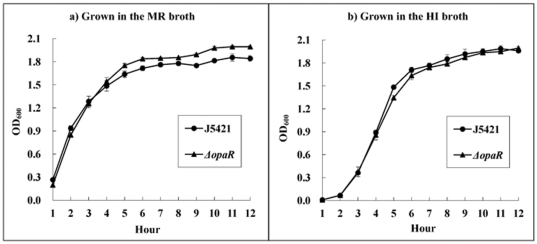
Growth curves. A two-round design of bacterial seed cultivation was employed: first, the glyceric stock of bacteria was inoculated into 15 ml of the MR or HI broth for growing at 30°C for 24 h with shaking at 200 rpm, and the cell culture was subsequently diluted to an OD_600_ value of about 1.0; second, the resulting culture was then 50-fold diluted into 15 ml of corresponding fresh MR or HI broth, and allowed to grow to reach an OD_600_ value of about 1.2 to 1.4. The bacterial seeds were 50-fold diluted into 15 ml of corresponding fresh MR or HI broth for further cultivation, and the OD_600_ values were monitored for each culture with a 1 h interval. Experiments were done in triplicate.

The growth curves of WT and *ΔopaR* grown at 30°C in the MR broth or in the HI broth were determined (Fig. 4). The two strains showed indistinguishable growth rates in each of the media. Thus, the *opaR* mutation had no effect on the bacterial *in vitro* growth.

For the following molecular regulation experiments, bacteria were pre-cultivated in the MR broth, spread onto grown on the HI agar plates for further growth, and harvested after an 8 h incubation at 30°C [at this status, the dense bacterial lawns (i.e. HCD) were observed on the agar]. It was thought that, unlike the liquid cultivation for which the autoinducers would disperse into the liquid media, the cultivation on solid medium would enable the enrichment of autoinducer molecules within the bacterial lawns with little dispersal into the agar.

### Negative auto-regulation of OpaR

**Figure 5:**
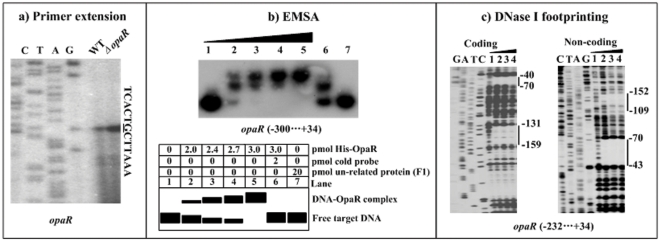
Repression of its own gene by OpaR. **a**) **Primer extension.** An oligonucleotide primer was designed to be complementary to the RNA transcript of *opaR*. The primer extension products were analyzed with 8 M urea-6% acrylamide sequencing gel. Lanes C, T, A, and G represent the Sanger sequencing reactions. The transcription start site of *opaR* was underlined in the DNA sequence. **b**) **EMSA.** The radioactively labeled DNA fragment from the 300th bp upstream to the 34th bp downstream of *opaR* was incubated with increasing amounts of purified His-OpaR protein, and then subjected to 4% (w/v) polyacrylamide gel electrophoresis. The band of free DNA disappeared with increasing amounts of His-OpaR protein, and a retarded DNA band with decreased mobility turned up, which presumably represented the DNA-OpaR complex. Shown on the lower side of the figure was the schematic representation of the EMSA design. **c**) **DNase I footprinting.** Labeled coding or non-coding DNA probes were incubated with increasing amounts of purified His-OpaR (Lanes 1, 2, 3, and 4 containing 0, 6, 9, and 12 pmol, respectively), and subjected to DNase I footprinting assay. Lanes G, A, T, and C represented the Sanger sequencing reactions. The footprint regions were indicated with vertical bars. The negative or positive numbers indicated the nucleotide positions upstream or downstream of *opaR*, respectively.

The primer extension experiments (Fig. 5a) were conducted to compare the yields of primer extension product of *opaR* in WT and *ΔopaR*. The primer extension assay detected a single transcription start site located at 74 bp upstream of *opaR*; therefore, a single promoter was transcribed for *opaR* under the growth condition tested. In addition, the *opaR* promoter activity was under the negative control of OpaR. A 334 bp promoter-proximal region of *opaR* was amplified, radioactively labeled, and subjected to EMSA with a purified His-OpaR protein (Fig. 5b). The results showed that His-OpaR was able to bind to this DNA fragment in a dose-dependent manner *in vitro*. As further determined by DNase I footprinting (Fig. 5c), the purified His-OpaR protein protected two distinct regions upstream of *opaR* against DNase I digestion in a dose-dependent manner. These two footprints, located from 70 to 40 bp (site 1) and from 159 to 109 bp (site 2) upstream of *opaR*, respectively, were considered as OpaR-binding sites. Taken together, OpaR is able to recognize the promoter of its own gene to directly repress its activity in *V. parahaemolyticus*.

### Stimulation of *qrr2–4* by OpaR

**Figure 6:**
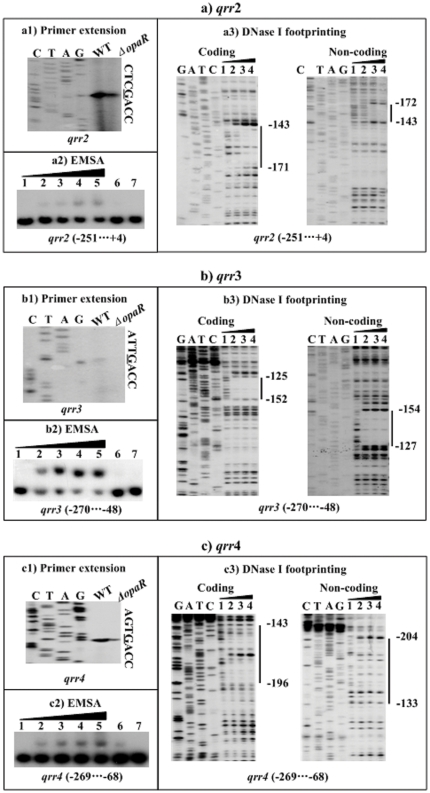
Stimulation *qrr2–4* by OpaR. For primer extension (a1, b1, and c1), an oligonucleotide primer was designed to be complementary to the RNA transcript of each of *qrr2–4*. For EMSA (a2, b2, and c2) and DNase I footprinting (a3, b3, and c3), the upstream DNA fragments of *qrr2–4* were radioactively labeled, and then incubated with increasing amounts of purified His-OpaR protein. The experiments were done as described in Fig. 5. The transcription start sites of *qrr2–4* were underlined in the DNA sequence. Lanes G, A, T, and C represented the Sanger sequencing reactions. The footprint regions were indicated with vertical bars. The negative or positive numbers indicated the nucleotide positions upstream or downstream of relevant *qr*r gene, respectively.

The primer extension assay (Fig. 6a) defined the transcription start sites the three sRNA genes *qrr2*–*4*, and this assay also indicated that the promoter activity of all the thee *qrr* genes was under the positive control of OpaR. Each of the promoter-proximal regions of *qrr2–4* was subjected to EMSA with the purified His-OpaR protein (Fig. 6b). The results showed that His-OpaR was able to bind to each of the three DNA fragments tested in a dose-dependent manner *in vitro*. As further determined by DNase I footprinting (Fig. 6c), His-OpaR protected a single region within each of the three upstream DNA fragments tested against DNase I digestion in a dose-dependent manner. These footprints, located from 172 to 143 bp, from 154 to 125 bp, and from 204 to 133 bp upstream of *qrr2–4*, respectively, were considered as OpaR-binding sites for these three genes. Taken together, OpaR is able to recognize the promoters of *qrr2–4* to activate their activity in *V. parahaemolyticus*.

### Repression of *aphA* by OpaR

**Figure 7:**
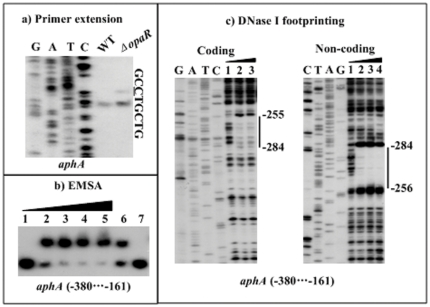
Repression of *aphA* by OpaR. For primer extension (a), an oligonucleotide primer was designed to be complementary to the RNA transcript of *aphA*. For EMSA (b) and DNase I footprinting (c), the DNA fragment from the 380th to 161th bp upstream of *aphA* was incubated with increasing amounts of purified His-OpaR protein. The experiments were done as described in Fig. 5. The transcription start site of *aphA* were underlined in the DNA sequence. Lanes G, A, T, and C represented the Sanger sequencing reactions. The footprint regions were indicated with vertical bars.. The minus numbers indicated the nucleotide positions upstream of *aphA*.

The primer extension assay (Fig. 7a) detected two closely neighboring extension products for *aphA*. Due to the facts that the shorter extension product might represent the premature stops due to the difficulty of polymerase in passing difficult sequences, and that the core promoter elements recognized by sigma factors could not be identified for the shorter extension product, only the longer product was chosen for the identification of the transcription start site that was located at 200 bp upstream of *aphA*. Therefore, a single promoter was transcribed for *aphA* under the growth condition tested, and its activity was under the negative control of OpaR. A 541 bp promoter-proximal region of *aphA* was subjected to EMSA with the purified His-OpaR protein (Fig. 7b). The results showed that His-OpaR was able to bind to the DNA fragment in a dose-dependent manner *in vitro*. As further determined by DNase I footprinting (Fig. 7c), His-OpaR protected a single region from 284 to 255 bp upstream of *opaR* against DNase I digestion in a dose-dependent manner. This footprint was considered as the OpaR-binding site for *aphA*. Taken together, OpaR is able to recognize the promoter of *aphA* to repress its activity in *V. parahaemolyticus*.

## Discussion

### Regulation of biofilm formation and virulence by *Vibrio* MQSRs

The *V. harveyi* LuxR mediates the repression of virulence determinant type III secretion system 1 (T3SS1) [31] and the stimulation of bioluminescence encoded by the *luxCDABEGH* operon [32]. LuxR binds to the upstream DNA region of *luxCDABEGH*, and thus stimulates this operon in a direct manner [32]; in contrast, LuxR indirectly represses the T3SS1 genes via directly repressing of *exsA*, a transcriptional activator of T3SS1, within the T3SS1 gene loci [31].

The *V. cholerae* HapR is a repressor of virulence: i) HapR directly represses the transcription of *aphA* [33] encoding a regulator [34] that in turns stimulates the expression of toxin-coregulated pilus; and ii) HapR inhibits the hemolytic activity at both transcriptional and posttranslational levels [35] (for the former mechanism, HapR directly represses the transcription of the hemolysin gene *hlyA*; for the later one, HapR directly stimulates the transcription of *hapA* encoding a metalloprotease that in turns degrades the HlyA hemolysin). HapR is also a repressor of biofim formation in *V. cholerae* [36]: i) HapR directly inhibits the transcription of *vpsT* encoding a transcriptional activator of biofilm formation; and ii) HapR represses the cellular c-di-GMP levels (c-di-GMP in turns acts as a posttranscriptional activator of the biofilm formation [37]) through directly modulating the transcription of multiple genes encoding GGDEF and EAL proteins.

The *V. vulnificus* SmcR is a repressor of cytotoxicity [38] that is important for the virulence of *V. vulnificus* and mainly dependent on two cytotoxins, RTX (encoded by *rtxA1*) and cytolysin/hemolysin (encoded by *vvhA*) [39], [40]. The transcription of *rtxA1* and *vvhA* is repressed by SmcR through directly repressing the transcription of *hlyU* [38], an activator of *rtxA1* and *vvhA* [40], [41]. SmcR-dependent expression of the metalloprotease gene *vvpE* is also found in *V. vulnificus* [42]; the two regulators cAMP receptor protein (CRP) and SmcR bind to the upstream region of *vvpE* in a juxtapositioned manner, and thus they function synergistically to coactivate the transcription of *vvpE* by the RpoS-dependent promoter at the stationary growth phase [42]. Whether the SmcR-dependent stimulation of metalloprotease contributes to the degradation of relevant protein toxins is still unclear in *V. vulnificus*.

The *V. parahaemolyticus* OpaR is a repressor of cytotoxicity to host cells [43], most likely through inhibiting the assembly/secretion of the cytotoxicity determinant T3SS1 [43], [44]. OpaR appears to repress biofilm formation through directly modulating the transcription of multiple genes encoding GGDEF and EAL proteins in pandemic O3:K6 *V. parahaemolyticus* (data unpublished). The molecular mechanisms employed by OpaR to regulate biofilm formation and virulence need to be elucidated.

Biofilm formation can be concisely linked to the bacterial survive in adverse conditions outside of the host, thus aiding in bacterial persistence during inter-epidemic seasons [45]. As shown in *V. cholerae* [45], [46], both intact and dispersed biofilms enhance the bacterial infectivity upon oral ingestion. Based on the previous speculations for *V. cholerae* [36], [47], [48], [49], a model of regulation of biofilm formation and virulence by QS during intestinal infection of pathogenic Vibrios is proposed herein: on the initial colonization (i.e., LCD) of a host, the expression of the MQSRs was inhibited by the Qrr sRNAs, and thus the expression of the biofilm formation and virulence genes occurs, which promotes the bacterial colonization and infection. When a HCD is reached, the MQSRs are abundantly produced, and thus inhibit biofilm formation and virulence in both direct (via control of structural genes) and indirect (via modulation of regulatory determinants) manners.

### Conserved *cis*-acting DNA signals recognized by MQSRs

Two consensus constructs, a box and a PFM, (Fig. 3) were built to represent the conserved *cis*-acting signals recognized by the four extremely conserved MQSR proteins, *V. harveyi* LuxR, *V. parahaemolyticus* OpaR, *V. vulnificus* SmcR, and *V. alginolyticus* ValR. These consensus constructs could be also applied to all the other members of the Harveyi clade in addition to the above four bacteria.

The 20 bp MQSR box was further annotated as an inverted repeat sequence. This dyad symmetry structure indicated that LuxR, OpaR, SmcR, and ValR, like other TetR-type proteins, bind to *cis*-acting regulatory DNA as a dimer. The box is a contiguous oligonucleotide, and thus it presents limited information that are originally presented in the MQSR-binding sites. Representation of the *cis*-acting regulatory motif with a PFM is able to give a much more comprehensive description of the uneven composition in each position, i.e., some nucleotides occurred much more frequently than others. Thus, the PFM presentedd here will over-represent the MQSR-binding sites more accurately than the 20 bp box sequence.

The PFM can be used to statistically predict the presence of MQSR consensus-like elements within the promoter-proximal sequences tested, which will generate a weight score for each gene, and a higher score denoted the higher probability of regulator-promoter association (Table 2). This assay was applied to the OpaR regulon members previously determined by microarray [43], when a frequently-used cutoff number of 7 was set for the score values, disclosing a set of candidates of direct OpaR targets (data not shown) in *V. parahaemolyticus* for further biochemical validation.

The PFM construct herein is essentially in agreement with those previously characterized for LuxR in *V. harveyi* [30] and SmcR in *V. vulnificus* [50]. A major difference is that the PFM of this study was constructed from the *cis*-acting DNA sequences from four closed related Vibrios, rather than from the artificial sequences [30], [50]. It was deemed that the PFM herein would enable the more accurate prediction of novel MQSR box-like sequences.

### Autoregulation of MQSRs

**Figure 8:**
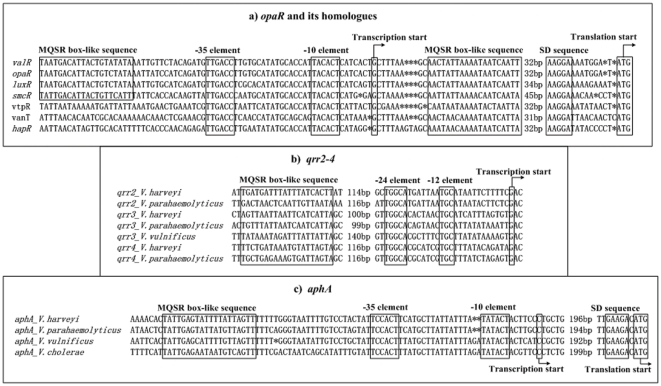
Organization of promoter DNA regions. DNA sequences were derived from *V. alginolyticus* ZJ-51 [60], *V. parahaemolyticus* RIMD 2210633 [16], *V. harveyi* ATCC BAA-1116 [61], *V. vulnificus* YJ016 [62], *V. tubiashii* RE22 [63], *V. anguillarum* 775 [64], *V. cholera* N16961 [65], and *V. fischeri* MJ11 [66]. Shown were translation and transcription starts, SD sequences, MQSR box-like sequences, and −10/−12 and −35/−24 core promoter elements.

Direct transcriptional repression of their own genes have been established for LuxR [29], HapR [51], VanT [52], and OpaR (this study). The promoter-proximal regions of *valR*, *opaR*, *luxR*, *smcR*, *vtpR*, *vanT*, and *hapR* were aligned in Fig. 8a, in which shown were translation and transcription starts, −35 and −10 core promoter elements for σ^70^ recognition, Shine-Dalgarno (SD) sequences for ribosome recognition, and MQSR box-like sequences representing the conserved signals for recognition by OpaR or its orthologs. This analysis gave a structural map of these auto-repressed promoters.

Two MQSR box-like sequences, upstream and downstream of the transcription start site, respectively, were annotated for *valR*, *opaR*, *luxR*, or *smcR*, indicating that two sites were recognized by the relevant regulatory protein for each target gene. Indeed, corresponding two binding sites have been experimentally determined for *opaR* (this study) or *luxR* [29]. Only one MQSR box-like sequence downstream of the transcription start site was annotated for *vtpR*, *vanT*, or *hapR*, indicating a single site within each of these promoter-proximal regions were recognized by the relevant regulatory protein; without no exception, a single corresponding HapR-binding site has been detected for *hapR* [51]. Notably, the MQSR box-like sequences downstream of the transcription start site were conservatively located within all the target promoter regions aligned, and the MQSR-promoter DNA association would block the entry of the RNA polymerase to repress the transcription of the target genes.

### Regulation of Qrr sRNAs genes by MQSRs

All of the three closely related organisms *V. harveyi*, *V. parahaemolyticus*, and *V. vulnificus* contain *qrr1–5*, and whereas, the more distantly related *V. cholerae* harbors only *qrr1–4*. Any one of Qrr1–4 in *V. cholerae* is perfectly sufficient to repress *hapR*, and thus, the four Qrr sRNAs are functionally redundant [2], [11]. Qrr1–4 but not Qrr5 are functional in *V. harveyi*, and Qrr5 may be an evolutionary vestige [4]. Unlike in *V. cholerae*, Qrr1–4 in *V. harveyi* function additively to control QS behaviors; these sRNAs function to translate increasing autoinducer concentrations (following the transition from LCD to HCD) into a precise gradient of LuxR protein, and that LuxR in turns induces a gradient of expression its target genes [4]. Functions of Qrr sRNAs in *V. parahaemolyticus* and *V. vulnificus* need to be elucidated.

As mentioned above, the LuxR- or OpaR-mediated stimulation of *qrr* transcription constitutes a negative feedback loop most likely maintaining the QS behaviors during the transition HCD to LCD [7], [8]. The *V. harveyi* LuxR directly binds to the upstream DNA regions of *qrr2–4* and stimulates the transcription of *qrr2–4*, but it has no regulatory effect on *qrr1* and *qrr5* [7]. In this work, we confirmed that *V. parahaemolyticus* OpaR also bound to the promoter regions of *qrr2–4* to activate their transcription. To the best of our knowledge, this is the first report of experimentally determining MQSR-binding sites and transcription starts of *qrr* genes. It should be noted the experimental transcription starts of *qrr* genes (this study) are 1 bp downstream of the predicted ones [2].

MQSR box-like sequences were found within the promoter-proximal regions of *qrr2–4* in *V. harveyi* and *V. parahaemolyticus*, and of *qrr3* in *V. vulnificus* (but not *qrr2* and *qrr4* of *V. vulnificus*). In addition, the box elements could not be predicted from *qrr1* and *qrr5* of all these three closely related Vibrios. These indicated that the MQSR-*qrr2–4* feedback loop was conversed between *V. harveyi* and *V. parahaemolyticus*. We aligned the promoter-proximal regions of *qrr2–4* (Fig. 8a) from *V. harveyi*, *V. parahaemolyticus*, and *V. vulnificus*, depicting translation and transcription starts, −24 and −12 core promoter elements for σ^45^ recognition, and MQSR box-like sequences (Fig. 8c). Since MQSR box-like sequences are upstream of the promoter −24 elements, the MQSR-stimulated *qrr* promoters may have a class I regulation that depends on the RNA polymerase α subunit C-terminal domain for function [53].

Unlike in *V. harveyi*, the *V. cholerae* HapR stimulates the transcription of all the *qrr1–4* in an indirect manner, since none of the binding of HapR to the *qrr* upstream regions can be detected [8]. As expected, MQSR box-like sequences could not be predicted from the upstream regions of all of *qrr1–4* in *V. cholerae*.

### Regulation of aphA by MQSRs

The AphA regulator is required for auto-repression [33], intestinal colonization and virulence [34], [54], biofilm formation [55], [56] in *V. cholerae*. In addition, as previously summarized [57], AphA and LuxR/HapR reciprocally control QS behaviors in *V. harveyi* and *V. cholerae*. At LCD, redundant Qrr sRNAs promote the AphA translation and meanwhile inhibit the LuxR translation. AphA further directly represses the *luxR* transcription, and also feeds back to repress the *qrr* transcription. At HCD, the cessation of Qrr sRNA production leads to no production of AphA, and the LuxR translation occurs. LuxR in turns directly represses the *aphA* transcription, and also feeds back to inhibit it own expression. Thus, AphA acts as a master regulator of QS behaviors at LCD, and in contrast, LuxR/HapR is the major one operating at HCD; the reciprocal gradients of AphA and LuxR/HapR are thought to be established for controlling the gene expression patterns during the transition between LCD and HCD [57].

The transcription of *aphA* is directly repressed by LuxR in *V. harveyi* [30] and by HapR *V. cholerae* [33], [48], and moreover HapR-binding site and transcription start have been determined for *aphA* in *V. cholerae* [48]. This study also detected the direct transcriptional repression of *aphA* by OpaR with the determination of transcription start and OpaR-binding site for *aphA* in *V. parahaemolyticus*. The alignment of the upstream regions of *aphA* from *V. harveyi*, *V. parahaemolyticus*, *V. vulnificus* and *V. cholerae* indicated that these four bacteria employed a conserved molecular mechanism for the repression of *aphA* by MQSRs, since transcription starts, −35 and −10 core promoter elements, SD sequences, and MQSR box-like sequences are conservatively located upstream of the *aphA* translation starts.

## Supporting Information

Figure S1 **Primer extension assay for validation of non-polar mutation.** The *opaR* null mutant *ΔopaR* was generated from the wild-type (WT) strain RIMD 2210633, and then the complemented mutant strain *C-opaR* was constructed. As determined by several distinct methods (see text), the transcription of *ahpA* was under the negative control of OpaR. Herein, an oligonucleotide primer, which was complementary to the RNA transcript of *ahpA*, was employed to detect the primer extension product that represented the relative mRNA level of *ahpA* in WT, *ΔopaR*, and *C-opaR*. The primer extension products were analyzed with 8 M urea–6% acrylamide sequencing gel. Lanes C, T, A, and G represented the Sanger sequencing reactions. The transcription start site (nucleotide C), which was located at 200 bp upstream of *ahpA*, was underlined in the DNA sequence. The *ahpA* mRNA level was significantly enhanced in *ΔopaR* relative to WT, while no obvious change in the *ahpA* transcription was observed between WT and *C-opaR*, which confirmed that the detecting enhanced transcription of *ahpA* in *ΔopaR* was due to the *opaR* mutation rather than a polar mutation.
(TIF)
