# Supplementary figures and images for: Transcriptional Regulation of opaR, qrr2–4 and aphA by the Master Quorum-Sensing Regulator OpaR in Vibrio parahaemolyticus
Source: PLoS One. 2012 Apr 10;7(4):e34622. doi: 10.1371/journal.pone.0034622 (PMC3323551; doi:10.1371/journal.pone.0034622)

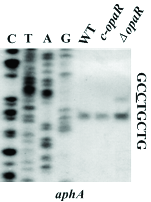

Supplement: Figure S1 — Primer extension assay for validation of non-polar mutation. The opaR null mutant ΔopaR was generated from the wild-type (WT) strain RIMD 2210633, and then the complemented mutant strain C-opaR was constructed. As determined by several distinct methods (see text), the transcription of ahpA was under the negative control of OpaR. Herein, an oligonucleotide primer, which was complementary to the RNA transcript of ahpA, was employed to detect the primer extension product that represented the relative mRNA level of ahpA in WT, ΔopaR, and C-opaR. The primer extension products were analyzed with 8 M urea–6% acrylamide sequencing gel. Lanes C, T, A, and G represented the Sanger sequencing reactions. The transcription start site (nucleotide C), which was located at 200 bp upstream of ahpA, was underlined in the DNA sequence. The ahpA mRNA level was significantly enhanced in ΔopaR relative to WT, while no obvious change in the ahpA transcription was observed between WT and C-opaR, which confirmed that the detecting enhanced transcription of ahpA in ΔopaR was due to the opaR mutation rather than a polar mutation. (TIF) [file pone.0034622.s001.tif]
